# Causal association of immune cells and polycystic ovarian syndrome: a Mendelian randomization study

**DOI:** 10.3389/fendo.2023.1326344

**Published:** 2023-12-22

**Authors:** Na Aru, Congyu Yang, Yuntian Chen, Jiaming Liu

**Affiliations:** ^1^ Department of Reproductive Endocrinology, West China Second University Hospital, Sichuan University, Chengdu, China; ^2^ Department of Radiology, West China Hospital, Sichuan University, Chengdu, China; ^3^ Department of Urology, West China Hospital, Sichuan University, Chengdu, China

**Keywords:** immune cells, causality, pcos, MR analysis, SNP

## Abstract

**Background:**

Polycystic ovarian syndrome (PCOS) is a common reproductive disorder that affects a considerable number of women worldwide. It is accompanied by irregular menstruation, hyperandrogenism, metabolic abnormalities, reproductive disorders and other clinical symptoms, which seriously endangers women’s physical and mental health. The etiology and pathogenesis of PCOS are not completely clear, but it is hypothesized that immune system may play a key role in it. However, previous studies investigating the connection between immune cells and PCOS have produced conflicting results.

**Methods:**

Mendelian randomization (MR) is a powerful study design that uses genetic variants as instrumental variables to enable examination of the causal effect of an exposure on an outcome in observational data. In this study, we utilized a comprehensive two-sample MR analysis to examine the causal link between 731 immune cells and PCOS. We employed complementary MR methods, such as the inverse-variance weighted (IVW) method, and conducted sensitivity analyses to evaluate the reliability of the outcomes.

**Results:**

Four immunophenotypes were identified to be significantly associated with PCOS risk: Memory B cell AC (IVW: OR [95%]: 1.123[1.040 to 1.213], *p* = 0.003), CD39+ CD4+ %CD4+ (IVW: OR [95%]: 0.869[0.784 to 0.963], *p* = 0.008), CD20 on CD20- CD38-(IVW: OR [95%]:1.297[1.088 to 1.546], *p* = 0.004), and HLA DR on CD14- CD16+ monocyte (IVW: OR [95%]:1.225[1.074 to 1.397], *p* = 0.003). The results of the sensitivity analyses were consistent with the main findings.

**Conclusions:**

Our MR analysis provides strong evidence supporting a causal association between immune cells and the susceptibility of PCOS. This discovery can assist in clinical decision-making regarding disease prognosis and treatment options, and also provides a new direction for drug development.

## Introduction

Polycystic ovary syndrome (PCOS) is a prevalent reproductive endocrine and metabolic disorder that commonly affects women of childbearing age. It is characterized by chronic anovulation and hyperandrogenism. Clinical symptoms of PCOS include menstrual irregularities, hirsutism, acne, and polycystic ovarian morphology. Additionally, individuals with PCOS may also experience metabolic conditions such as obesity, insulin resistance, and dyslipidemia (1). The pathogenic mechanisms of PCOS remain unclear; in addition to the ovarian-pituitary-hypothalamic-gonadal axis, pathogenesis of PCOS must also consider ovarian local cytokines, immunology, and genetics.

Recent research has highlighted the importance of the inflammatory immune mechanism in the occurrence and development of PCOS. Numerous studies have reported that chronic low-grade inflammation is closely associated with and interacts with PCOS (2–4). The identification of leukocytosis in polycystic ovaries may indicate that polycystic ovaries are associated with a pro-inflammatory state (5, 6). The expression of IFN-c, a cytokine produced by Th1, was significantly increased in PCOS patients compared to the control group (3). The regulation of granulosa cells and immune cells is impaired in patients with PCOS, which may contribute to accelerated anovulation (7). Systemic and ovarian cytokines, such as tumor necrosis factor (TNF)-α, interleukin (IL)-6, and IL-18, can alter the local microenvironment in the ovary, disrupt ovarian function, increase androgen production, and contribute to insulin resistance through various mechanisms (2). Immune factors such as vascular endothelial growth factor (VEGF) and transforming growth factor-β1, along with inflammation in the follicular microenvironment, may play a role in the dysfunction of the hypothalamic-pituitary-gonadal axis and the development of follicular dysplasia. Patients with PCOS had higher levels of antinuclear antibody, histone antibody resistance, and ds-DNA antibody levels than the control group. In addition, an increase in thyroperoxidase or thyroglobulin antibodies in patients with PCOS was found to be associated with the development of autoimmune thyroiditis (8).

Wu et al. found that T lymphocytes play a significant role in the local pathological mechanisms of PCOS (9). T lymphocytes secrete inflammatory and immunomodulatory molecules that regulate ovarian function. Additionally, dysregulation of T-cell subsets has been observed in the peripheral blood and ovaries of patients with PCOS due to disrupted sex hormone levels (10). Animal models with elevated androgens have been linked to reproductive dysfunction, including oligo-anovulation, menstrual disturbances, and subfertility, which are commonly observed in PCOS (11–15). Androgens have immunomodulatory effects, and the presence of elevated androgens is associated with altered immune function, which can have an impact on reproductive function (16, 17). Medawar identified the importance of the immune system in reproduction, and subsequent studies have highlighted the significance of regulatory T (Treg) cells frequencies in maintaining normal ovarian function and menstrual cycles (18–20). Androgens appear to modulate the differentiation of T cells and the ratios of Treg cells (3, 17, 21, 22). Furthermore, the differentiation of T cells is also modulated by epigenetic mechanisms (23–25), which may be the case in PCOS (17).

PCOS has been shown to be significantly associated with B lymphocytes. In addition to their role in humoral immunity, B lymphocytes are involved in antigen detection and regulation of antigen processing and presentation. This group of lymphocytes plays a crucial role in the development of insulin resistance associated with obesity and glucose intolerance. They contribute to insulin resistance by activating pro-inflammatory T cells and producing pathological antibodies (26). The pathogenic role of B cells was identified in PCOS, as the activity of these cells increased in women with PCOS compared to the control group. Moreover, mice with PCOS that were treated with CD19 antibody exhibited a reduction in B cells in their peripheral blood, which led to a reduction in their cystic follicles and an increase in their corpus luteum. Based on these findings, it can be concluded that manipulation of these cells and antibodies could be potential targets for treating insulin resistance and PCOS (27).

Mendelian randomization (MR) is an analytical method used in epidemiological etiology inference, which is based on the Mendelian independent distribution law. It is essential for the causal sequence of MR to be reasonable (28, 29). Previous observational studies have identified multiple associations between immune cell traits and PCOS, supporting the hypothesis of a correlation between them. In this study, a comprehensive two-sample MR analysis was conducted to establish a causal association between immune cell signatures and PCOS.

## Materials and methods

### Study design

We assessed the causal relationship between 731 immune cell signatures and PCOS based on a two-sample MR analysis. In order to acquire dependable outcomes, three hypotheses must be met during the execution of MR analysis: a robust association between genetic variants and exposure factors, an absence of correlation between genetic variants and confounding variables, and the influence of genetic variants on the outcome solely through exposure factors, excluding other pathways.

### Genome-wide association study data sources for PCOS

The GWAS statistics for PCOS were sourced from FinnGen Research’s data release in July 2021 (https://gwas.mrcieu.ac.uk/datasets/finn-b-E4_POCS/). The diagnostic criteria of PCOS were based on ICD-9 and ICD-10 standards (presence of two of the three criteria: chronic anovulation, hyperandrogenism, polycystic ovaries on ultrasonography), and the GWAS statistics encompassed 16,379,676 loci variations from 642 cases and 118,228 controls.

### Immunity-wide GWAS data sources

The GWAS summary statistics for each immune trait can be accessed from the GWAS Catalog, with accession numbers ranging from GCST90001391 to GCST90002121 (30). A total of 731 immunophenotypes were included in the analysis, which comprised of absolute cell (AC) counts (n = 118), median fluorescence intensities (MFI) representing surface antigen levels (n = 389), morphological parameters (MP) (n = 32), and relative cell (RC) counts (n = 192). The AC, MFI, and RC features encompassed B cells, CDCs, mature stages of T cells, monocytes, myeloid cells, TBNK (T cells, B cells, natural killer cells), and Treg panels. The MP feature consisted of CDC and TBNK panels. The original GWAS on immune traits utilized data from 3,757 European individuals, and there were no overlapping cohorts. Approximately 22 million SNPs were genotyped with high-density arrays and imputed using the Sardinian sequence-based reference panel (31). Associations were tested while adjusting for covariates such as sex, and age.

### Selection of instrumental variables

Based on recent research (30), the significance level for instrumental variables (IVs) associated with each immune trait was set to 1 × 10^-5^. To ensure reliable results, a threshold for strong linkage disequilibrium (LD) effect was applied (r^2^ < 0.001) (32), with LD r^2^ calculated using the 10000 Genomes Project as a reference panel. The proportion of phenotypic variation explained (PVE) and F statistic were calculated for each IV to assess IV strength and avoid weak instrumental bias. Furthermore, to mitigate bias introduced by weak instruments, IVs with F statistics greater than 10 were deemed strong instruments and retained for subsequent analysis. The exposure and outcome SNPs were harmonized to align effect estimates for the same effect allele. Palindromic SNPs with intermediate effect allele frequencies (EAFs > 0.42) or SNPs with incompatible alleles were excluded (33).

### Data analysis

We conducted a range of MR analyses, encompassing MR Egger, weighted median, inverse-variance weighted (IVW), simple mode, weighted mode, and MR-PRESSO approaches. Among these, the IVW method is frequently employed (34).

To evaluate the presence of variance, we performed heterogeneity examinations utilizing both the MR Egger and IVW techniques. The Cochrane’s Q value was employed to appraise the variability of genetic instruments, whereby a p-value exceeding 0.05 indicates a lack of noteworthy diversity. To scrutinize the existence of horizontal pleiotropy, we utilized the MR Egger regression equation, where a p-value surpassing 0.05 indicates an absence of indications of horizontal pleiotropy (35).

**Figure 1:**
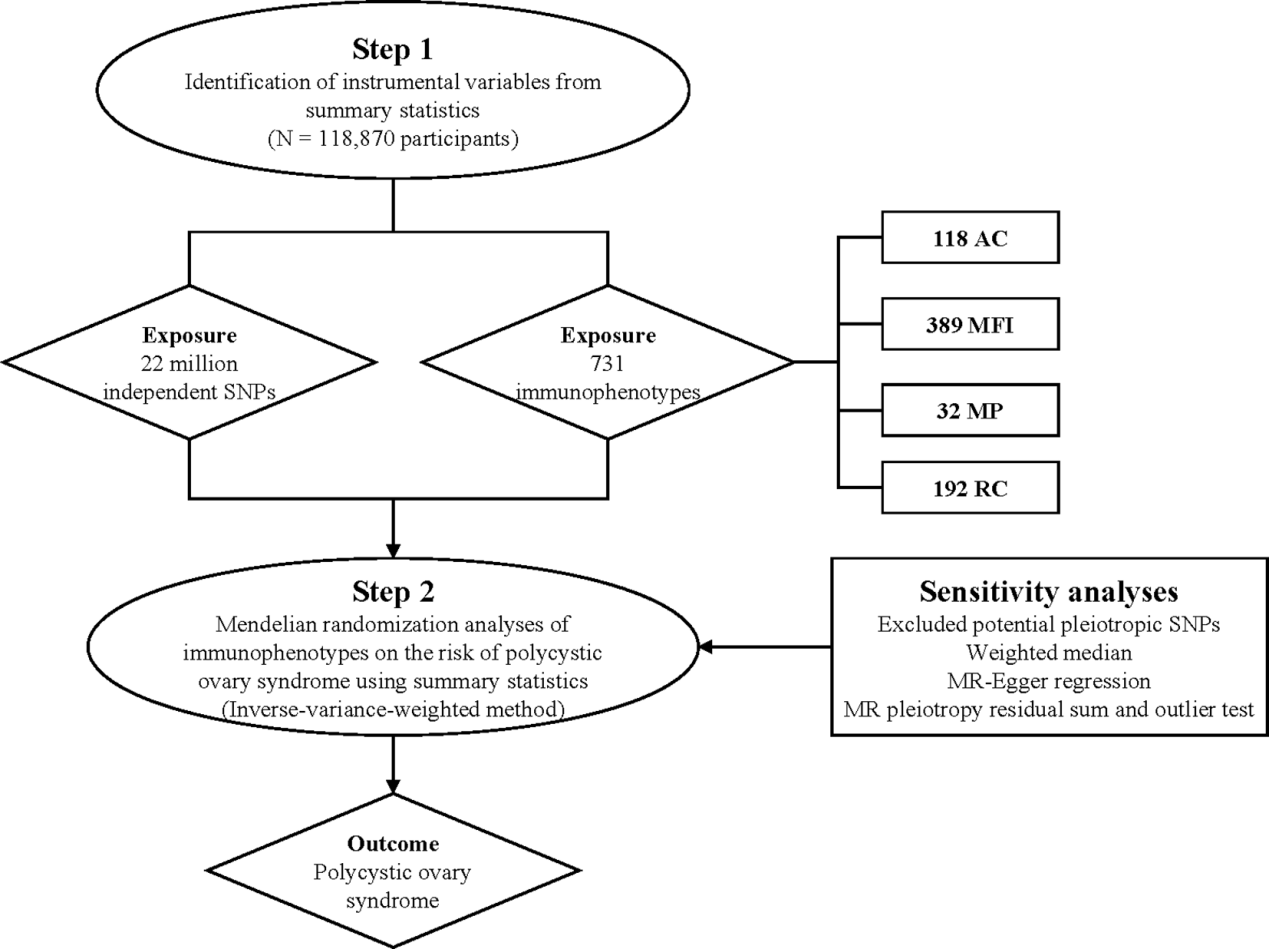
Research overview and design of Mendelian randomization analysis.

Additionally, in order to assess the potential impact of directional pleiotropy, we scrutinized each SNP for potential associations with secondary phenotypes using the GWAS Catalog (http://www.phenoscanner.medschl.cam.ac.uk/). Subsequently, we re-performed the MR analyses, excluding SNPs associated with other phenotypes. Moreover, we conducted leave-one-out sensitivity analyses on significant findings to determine if a single SNP was accountable for the observed causal relationship. The overall research design is depicted in **Figure 1**. The MR analyses were carried out utilizing the ‘TwoSampleMR’ package (version 0.5.7) within the R software environment (version 4.2.1) (35, 36).

## Results

### Exploration of the causal effect of immunophenotypes on PCOS

At the significance of 0.001, we detected Memory B cell AC, CD20 on CD20- CD38-, HLA DR on CD14- CD16+ monocyte were significantly associated with an increased risk of PCOS, while CD39+ CD4+ %CD4+ retained a robust association with an decreased risk of PCOS.

**Figure 2:**
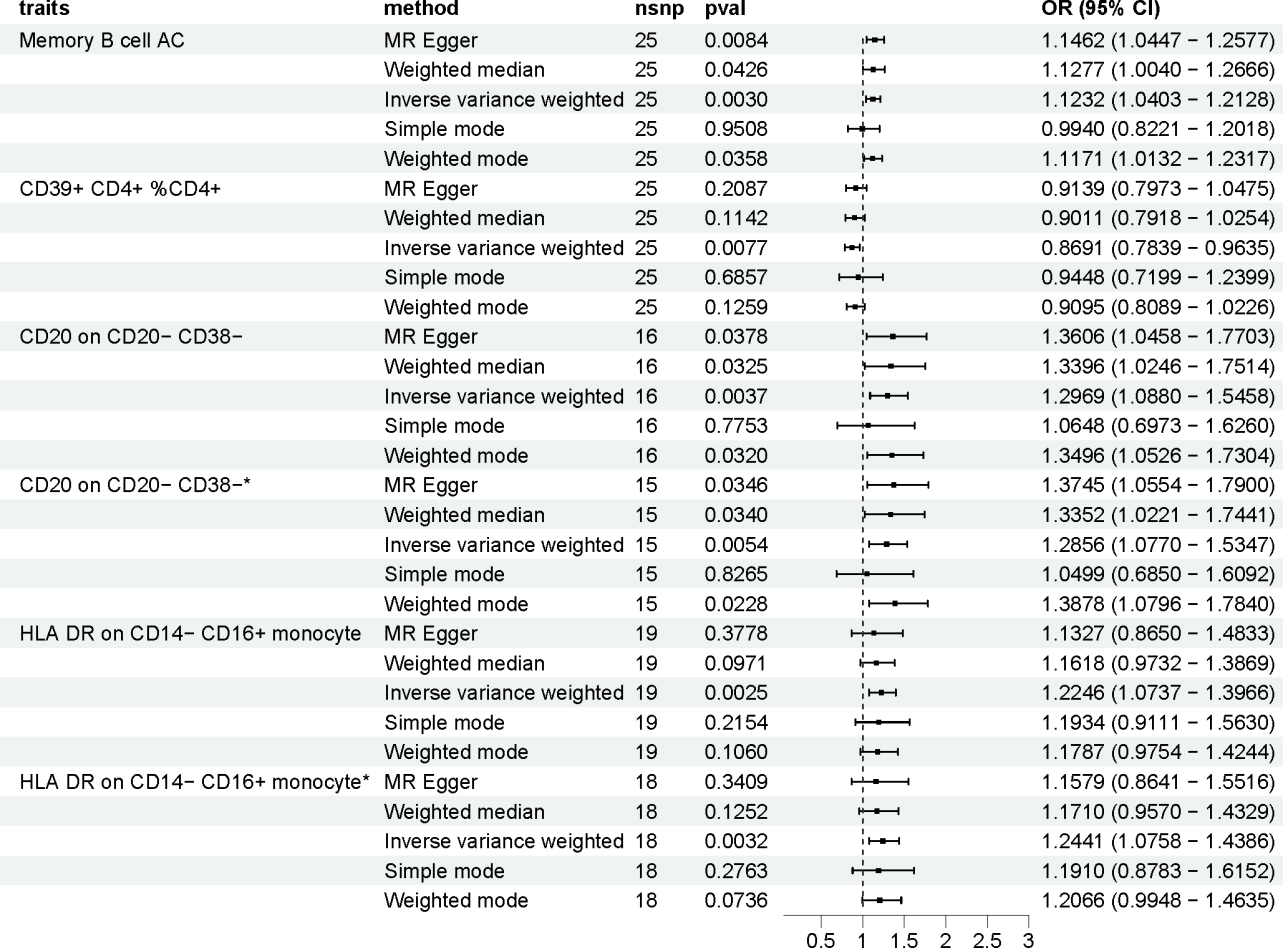
The effect of immune cells on polycystic ovarian syndrome. Asterisk (*) represents MR analysis results after excluding SNPs associated with other phenotypes. nsnp, number of single nucleotide polymorphisms; OR, odds ratio; CI, confidence interval.

The OR of Memory B cell AC (B cell panel) on PCOS risk was estimated to be 1.123 (95% CI = 1.040 to 1.213, *p* = 0.003) by using the IVW method. Similar results were observed by using MR-Egger (OR [95%]: 1.146 [1.045 to 1.258], *p* = 0.008). The genetically predicted CD39+ CD4+ %CD4+ (Treg panel) exhibited a noticeable protective effect against PCOS (IVW: OR [95%]:0.869 [0.784 to 0.963], *p* = 0.008). The genetically predicted CD20 on CD20- CD38- (B cell panel) showed a positive correlation with the risk of PCOS, as evidenced by IVW method (OR [95%]:1.297 [1.088 to 1.546], *p* = 0.004). The OR of HLA DR on CD14- CD16+ monocyte (Monocyte panel) on PCOS risk was estimated to be 1.225 (95% CI = 1.074 to 1.397, *p* = 0.003) by using the IVW method. (**Supplementary Table 1**, **Figure 2**).

### Sensitivity analysis

The MR-Egger intercept test and MR-PRESSO global test results indicated no evidence of heterogeneity or horizontal pleiotropy in the associations between immunophenotypes and PCOS. These results are presented in **Supplementary Table 2**. Furthermore, the leave-one-out analysis demonstrated the robustness of the MR results. Excluding any single SNP associated with immunophenotypes and PCOS did not significantly alter the overall findings.

To account for potential directional pleiotropy, we conducted an analysis using the GWAS Catalog to identify SNPs linked to immunophenotypes and PCOS. Two SNPs were discovered to exhibit associations with other traits, as detailed in **Supplementary Table 3**. After excluding these pleiotropic SNPs, the associations between immunophenotypes and PCOS remained stable, as shown in **Supplementary Table 4**.

## Discussion

Our study integrates large-scale individual and aggregated GWAS datasets to systematically elucidate the role of immune cells in the pathogenesis of PCOS from a genetic perspective. Our analysis provides suggestive evidence that immune cells can influence the risk of PCOS through a comprehensive genetic approach based on large-scale GWAS summary data. To the best of our knowledge, this is the first MR analysis to investigate the causal relationship between multiple immunophenotypes and PCOS. By utilizing SNPs as instrumental variables and integrating various two-sample MR methods, we confirmed that four immune cells, including Memory B cell AC, CD39+ CD4+ %CD4+, CD20 on CD20- CD38- and HLA DR on CD14- CD16+ monocyte, were significantly associated with the risk of PCOS.

Our findings indicate that two types of B cells, namely CD20 on CD20- CD38- and Memory B cell AC, were significantly associated with an increased risk of PCOS. Recent studies have highlighted the important role of B cells in the development of PCOS. B lymphocytes are known to produce antibodies in response to self-antigens, and the formation of antigen-antibody complexes can contribute to inflammatory responses in the body, thereby potentially increasing the risk of PCOS (37). CD20 is a distinct antigen found on the surface of B lymphocytes, and it is recognized for its significant involvement in regulating B lymphocyte proliferation, differentiation, and signaling processes (38). Previous studies have characterized Memory B cells as being enriched with autoantibodies and primed for plasma cell differentiation. They have also been associated with excessive accumulation in chronic infections, autoimmune disorders, and immunodeficiencies, suggesting their involvement in the regulation of humoral responses. Consistent with existing data demonstrating increased B cell frequencies in PCOS, hyperandrogenic women with PCOS showed a significant reorganization of their B cell repertoire, leading to elevated frequencies of B memory cells (39).

Our study revealed a correlation between elevated levels of CD39+ CD4+ %CD4+ (Treg panel) and a decreased risk of PCOS. T cells can be categorized into three subsets: T helper cells, Cytotoxic T cells, and Treg cells. Treg cells are crucial for immune system regulation, homeostasis, and prevention of autoimmunity. Previous research on Treg cell proliferation in PCOS patients has shown a decrease in these cells (40). Additionally, studies have indicated reduced levels of anti-inflammatory factors like IL-10 in the bodies and ovaries of PCOS patients, attributed to a reduction in peripheral blood Treg cells (41). Treg cells CD39+ CD4+ %CD4+ are particularly important for reproductive function. During a normal pregnancy, there is an increase in the number of Treg cells CD39+ CD4+ % CD4+, while studies suggest that a decrease in these cells among PCOS patients could contribute to miscarriage or infertility (42).

In our study, we discovered a correlation between HLA DR on CD14- CD16+ monocyte levels and an increased risk of PCOS. Androgens can disrupt the ovarian immune balance in PCOS by interacting with immune cells and cytokines. Research revealed that monocytes entering the ovary can provoke a local inflammatory response, leading to increased production of ovarian androgens in women with PCOS (43). Tumor necrosis factor-alpha (TNF-α), released by monocytes, has been associated with insulin resistance in PCOS (43). Monocytes and macrophages act as immune sentinels in the innate immune system and can be distinguished by their expression of CD14 and CD16. Monocyte subsets are classified based on phenotypic markers: classic (CD14+CD16-), intermediate (CD14+CD16+), and nonclassical (CD14−CD16+). Our findings have demonstrated the involvement of non-classical monocytes in PCOS. Non-classical monocytes secrete a significant amount of IL-1β in a TLR signaling-dependent manner (44). Compared to classical monocytes, the CD16+ subset exhibits a stronger ability to release pro-inflammatory factors and, as a result, is increased in individuals with PCOS (45, 46).

It is necessary to acknowledge that our study possesses certain inherent limitations that cannot be overlooked. Firstly, it is important to recognize that MR analysis cannot serve as a substitute for clinical trials within the objective realm, as it merely serves as a method for analyzing the causal relationship between exposure and outcome. Therefore, further investigations are required to corroborate the potential association between immune cells and the risk of PCOS. Additionally, our MR analysis was exclusively conducted within the European population due to the limited availability of GWAS data resources. Given the genetic heterogeneity among various ethnic groups, results may vary across different populations. Consequently, forthcoming studies should undertake subgroup analyses encompassing diverse populations in order to arrive at a more comprehensive and encompassing conclusion.

In conclusion, our MR analysis results indicate that Memory B cell AC, CD20 on CD20- CD38-, HLA DR on CD14- CD16+ monocyte increase the risk of PCOS, while CD39+ CD4+ %CD4+ may lead to decreased risk of PCOS. This discovery can assist in clinical decision-making regarding disease prognosis and treatment options, and also provides a new direction for drug development. However, the pathogenesis of PCOS is multifaceted, and the clinical heterogeneity of various types of immune cells involved in PCOS is evident. Therefore, a single treatment may not always yield the desired outcomes. Further research is needed to investigate the interplay between innate immune cells and between innate and adaptive immune cells in PCOS patients.

## Data availability statement

The datasets presented in this study can be found in online repositories. The names of the repository/repositories and accession number(s) can be found in the article/**Supplementary Material**.

## Ethics statement

The studies involving humans were approved by The GWAS summary data used in this study were all from the online public platform (https://gwas.mrcieu.ac.uk/). The study protocols were approved by respective local ethics committees, and participants have provided written informed consent. The studies were conducted in accordance with the local legislation and institutional requirements. Written informed consent for participation was not required from the participants or the participants’ legal guardians/next of kin in accordance with the national legislation and institutional requirements.

## Author contributions

NA: Writing – original draft. CY: Investigation, Writing – review & editing. YC: Data curation, Writing – review & editing. JL: Funding acquisition, Supervision, Writing – review & editing.
